# Comparison between primary repair and augmented repair with gastrocnemius turn-down flap for acute Achilles tendon rupture: a retrospective study with minimum 2-year follow-up

**DOI:** 10.1186/s12891-023-06260-w

**Published:** 2023-03-04

**Authors:** Shuai Yang, Weili Shi, Wenqiang Yan, Yingfang Ao, Qinwei Guo, Yuping Yang

**Affiliations:** Department of Sports Medicine, Peking University Third Hospital, Institute of Sports Medicine of Peking University, Beijing Key Laboratory of Sports Injuries, Engineering Research Center of Sports Trauma Treatment Technology and Devices, Ministry of Education, 49 North Garden Road, Beijing, Haidian District 100191 China

**Keywords:** Achilles tendon rupture, Acute injury, Primary repair, Augmented repair, Gastrocnemius turn-down flap

## Abstract

**Background:**

To explore and compare the clinical outcomes in patients undergoing primary repair versus augmented repair with a gastrocnemius turn-down flap for acute Achilles tendon rupture.

**Methods:**

From 2012 to 2018, the clinical data of 113 patients with acute Achilles tendon rupture who were treated with primary repair or augmented repair with a gastrocnemius turn-down flap by the same surgeon were retrospectively reviewed. The patients’ preoperative and postoperative scores on the visual analog scale (VAS), American Orthopaedic Foot and Ankle Society Ankle⁃Hindfoot (AOFAS) score, the Victorian Institute of Sport Assessment⁃Achilles (VISA-A), the Achilles tendon total rupture score (ATRS), and the Tegner Activity Scale were examined and compared. The postoperative calf circumference was measured. A Biodex isokinetic dynamometer was used to evaluate the plantarflexion strength on both sides. The time to return to life and exercise as well as the strength deficits in both groups were recorded. Finally, the correlation analyses between patient characteristics and treatment details with clinical outcomes were conducted.

**Results:**

In total, 68 patients were included and completed the follow-up. The 42 and 26 patients who were treated with primary repair and augmented repair were assigned to group A and B, respectively. No serious postoperative complications were reported. No significant between-group differences in any outcomes were observed. It was found that female sex was correlated with poorer VISA-A score (*P* = 0.009), complete seal of paratenon was correlated with higher AOFAS score (*P* = 0.031), and short leg cast was correlated with higher ATRS score (*P* = 0.006).

**Conclusions:**

Augmented repair with a gastrocnemius turn-down flap provided no advantage over primary repair for the treatment of acute Achilles tendon rupture. After surgical treatment, females tended to had poorer outcomes, while complete seal of paratenon and short leg cast contributed to better results.

**Level of evidence:**

Cohort study; Level of evidence, 3.

## Introduction

Acute Achilles tendon rupture is a common form of acute lower-extremity trauma that is particularly prevalent among men aged 30 to 50 years. The incidence of Achilles tendon rupture has increased from 17.3 to 32.3 per 100,000 person-years during the last decade [1]. Risk factors have included, but not limited to, corticosteroids, fluoroquinolones, and repetitive microtrauma [2]. The common site for the Achilles tendon rupture is 2 to 6 cm proximal to the calcaneus insertion. Narrow cross-sectional area and low blood supply are two reasons that Achilles tendon is prone to injuries and difficult to heal [3, 4].

The optimal treatment for acute Achilles tendon rupture is the subject of intense debate [5–17]. Although conservative management has shown similar functional results with surgery and a growing trend recently, the surgical therapy remains an important option for acute Achilles tendon rupture [4]. Open surgery, which involves alignment and suturing of the torn ends of tendon under direct vision, is reliable and enables patients to undergo more active rehabilitation, resulting in a faster recovery and lower risk of rerupture [5, 18–20]. The most common types of open surgery are end-to-end primary repair and augmented repair, of which the former has higher tensile strength while the latter is favored for the shorter incision and lower risk of wound problems [9]. However, few studies have compared the clinical outcomes of primary repair and augmented repair with gastrocnemius turn-down flap [21].

The purpose of the present retrospective study was to compare the recovery outcomes of patients with acute Achilles tendon rupture undergoing primary repair versus augmented repair with a gastrocnemius turn-down flap, and to further distinguish factors associated with clinical outcomes. Our hypothesis was that the clinical outcomes in patients receiving augmented repair were not superior to that receiving primary repair. Additionally, complete seal of paratenon and short leg cast were hypothesized to lead to better clinical results.

## Methods

### General data

Patients who received a diagnosis of acute Achilles tendon rupture and underwent surgical treatment within 2 weeks of injury, had difficulty standing on 1 foot and raising their heel, were followed up for at least 2 years, and had an age of 18–50 years were included. Patients with open wounds, Achilles tendon enthesopathy, a history of topical steroid injections, other conditions such as fractures or damage to the nerves and blood vessels, as well as rerupture were excluded.

The data of 113 patients with acute Achilles tendon rupture who underwent surgical repair by the same surgeon were retrospectively reviewed from April 2012 to April 2018. Forty-five patients were excluded due to lost to follow-up (*n* = 31), participation decline (*n* = 5), follow-up less than 2 years (*n* = 4), age more than 50 years (*n* = 4), age less than 18 years (*n* = 1). A final total of 68 patients (64 men and 4 women) were included, with an average age of 35 years (range 20–49 years).

The 42 patients who received primary repair (using end-to-end suture) were assigned to group A, and the 26 patients who received augmented repair with a gastrocnemius turn-down flap (a procedure that was based on end-to-end suture) were assigned to group B. No significant differences in sex, age, medical history, body mass index, side of injury, and follow-up duration were observed (Table 1).

**Table 1:** Patient demographics

Characteristic	Group A	Group B	*P* Value
Sex (male/female)	39/3	25/1	0.574
Age(y)	35.5 ± 7.4	34.8 ± 6.5	0.699
Time to presentation (d)	4.3 ± 2.2	4.3 ± 2.5	0.883
Body mass index (kg/m^2^)	25.8 ± 3.4	25.8 ± 3.0	0.962
Side (left/right)	25/17	18/8	0.420
Follow-up period (mo)	51.7 ± 18.9	52.3 ± 12.8	0.873

### Surgical methods

**Fig. 1:**
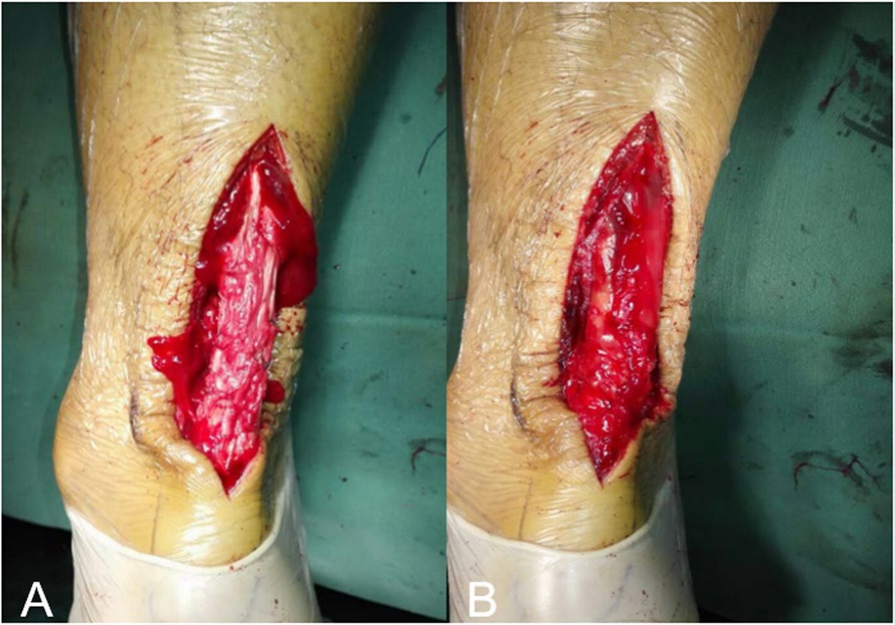
Operative photograph of paratenon seal. **A** The torn ends were opposed and repaired, leaving the paratenon unsealed; **B** The paratenon was completely sealed

All the operations were conducted by the same surgeon. The decision to use which technique was made by the surgeon and patients’ preference after they were informed of the benefit and risk of two techniques. During all operations, patients were in prone position, spinal anesthesia and lower-extremity tourniquets were applied. A vertical incision of 6 to 8 cm was made on the inside of the Achilles tendon rupture. The skin, subcutaneous tissue, and deep fascia were incised layer by layer until the torn ends of the Achilles tendon were revealed. Subsequently, the blood clots were cleaned, and the frayed tendon ends were carefully debrided. Specifically, in group A, the torn ends were pulled using a hemostat and aligned, and overlapping repair was performed using 2–0 Vicryl antibacterial absorbable sutures (Johnson & Johnson, USA). In group B, on the basis of primary repair, flaps of the proximal gastrocnemius (length, width, and thickness: 6 to 8, 1 to 1.5, and 2 to 3 mm, respectively) were removed. The flaps were then turned down and secured with intermittent sutures (using the same 2–0 absorbable sutures) on both sides and at the center to eliminate dead spaces. In both groups, the surgeon ensured that the calcaneal tubercle was the same height as its contralateral side. The surgical wound was then washed, and the intact paratenon was completely sealed using 3–0 absorbable sutures. However, in patients with incomplete paratenon structure, the paratenon could not be completely sealed (Fig. 1). Each layer was sealed using intermittent sutures (again with absorbable sutures). After surgery, a compressive dressing was applied, and the limb was immobilized with a long leg cast keeping the ankle in natural plantar flexion. Later in the study, some patients underwent short leg cast immobilization immediately after operation [22].

### Postoperative rehabilitation

We encouraged the patients to initiate rehabilitation (e.g., toe- and leg-lifting exercises) that did not substantially aggravate their pain as soon as possible. Two weeks postoperation, all the patients underwent short leg cast immobilization and began knee joint exercises. Four weeks postoperation, ankle exercises started to increase proprioception, plantar flexion, inversion and eversion. Six weeks postoperation, patients were instructed to begin partial weight-bearing exercise. Using a heel pad with a thickness between 3 and 3.5 cm, they practiced walking on crutches. Twelve weeks postoperation, the heel pad was replaced with one of 2 cm in thickness, and the patients began taking full weight-bearing walks without crutches. Sixteen weeks postoperation, the heel pad was removed, enabling the patients to practice walking normally, with gradual improvement. Twenty weeks postoperation, they could begin low-impact exercise. Twenty-eight weeks postoperation, according to their recovery situation, the surgeon determined when they could begin recreational sports.

### Evaluation on the clinical outcomes

The patients were followed up during outpatient visits and were contacted to complete online questionnaire surveys at last follow-up. The clinical follow-up was performed by two independent observers who were not involved in surgical treatment. Before operation, all the patients received face-to-face training on questionnaire survey to ensure maximum scoring accuracy. A visual analog scale was used for the pain assessments. The function and activity level of the Achilles tendon were examined using the American Orthopaedic Foot and Ankle Society ankle-hindfoot scale, the Victorian Institute of Sport Assessment⁃Achilles scale, and the Achilles tendon total rupture score. Lower-extremity activity was evaluated via the Tegner Activity Scale.

At 3 months postoperation, the patients underwent isokinetic testing of calf plantarflexion and muslce strength on a dynamometer (Biodex Medical Systems, Shirley, New York). We obtained the peak torque (N·m) when patients were extending the ankle joint in the speed 60 deg/sec and 120 deg/sec and then calculated the deficit percentage between the affected and unaffected ankle. The deficit percentage = 1- (Peak torque in the affected side) / (Peak torque in the unaffected side). Muscle performance was also evaluated with the ability to perform a single-leg heel rise (at least 30 raises per minute and height of the heel should be at least 5 cm from the floor).

### Statistical analysis

All analyses were conducted using IBM SPSS Statistics for Windows, version 24 (IBM Corp., Armonk, NY, USA). A chi-squared test was performed on the patients’ general characteristics and conditions (e.g., sex and side of injury). One-way analysis of variance was used to compare the medical history and follow-up duration of the two groups. Mann–Whitney test was performed to examine between-group differences in physical function scores before and after the surgery. Bivariate analysis was performed using Spearman’s rank correlation coefficients for correlations between patient characteristics and treatment details (age, sex, body mass index [BMI], time to presentation, complete seal of paratenon, length of leg cast) with all final clinical outcomes. A *P* value of less than 0.05 was considered statistically significant.

## Results

As shown in table 2, no significant differences in all the postoperative clinical functional scores could be observed between two groups. In the last follow-up session, all the patients were able to perform a single-leg calf raise without pain and had regained their ability to perform activities of daily life or exercises, with no significant difference of the mean recovery time between two groups. In addition, no significant between-group differences were noted in the isokinetic testing results at 3 months postoperation, same as calf circumference at 3 and 6 months postoperation. However, the calf circumference on the operated side was less than the contralateral side in either group, both at 3 and 6 months postoperatively. No serious complications (e.g., deep infections and gastrocnemius nerve damage) or Achilles tendon re-rupture were noted in either group.

**Table 2:** Clinical Outcomes After surgery

	Group A	Group B	*P* Value
VAS	0.2 ± 1.1	0.3 ± 1.4	0.620
AOFAS	98.5 ± 6.3	99.1 ± 2.1	0.790
VISA-A	94.0 ± 9.3	93.0 ± 4.7	0.061
Tegner	5.0 ± 1.5	5.0 ± 1.3	0.447
ATRS	97.7 ± 4.5	97.5 ± 5.4	0.679
Time to return to daily life(m)	3.5 ± 1.4	3.1 ± 1.0	0.315
Time to return to non-weight-bearing exercise(m)	9.2 ± 4.3	8.4 ± 4.6	0.214
Time to return to weight-bearing exercise(m)	11.2 ± 5.1	9.8 ± 4.7	0.304
Plantar flexion peak torque deficits at 3 months post-surgery			
60°	0.3 ± 0.8	0.2 ± 0.6	0.632
120°	0.1 ± 0.2	0.2 ± 0.2	0.765
Calf circumference at 3 months post-surgery			
Operated side	37.9 ± 2.2	36.8 ± 3.9	0.524
Contralateral side	39.5 ± 2.6	39.2 ± 3.0	0.522
P Value	0.011	0.043	
Calf circumference at 6 months post-surgery			
Operated side	38.8 ± 2.3	38.4 ± 3.1	0.953
Contralateral side	40.3 ± 3.1	39.7 ± 2.2	0.813
P Value	0.002	0.002	

*VAS* Visual analogue scale; *AOFAS* American Orthopaedic Foot and Ankle Society Ankle⁃Hindfoot Score; *VISA-A* the Victorian Institute of Sport Assessment⁃Achilles; *ATRS* the Achilles tendon total rupture score; *Tegner* the Tegner Activity Scale

During both surgical approaches, the surgeon sealed the paratenon to the greatest extent to reduce the risk of postsurgical infections. However, in patients with incomplete paratenon structure, the paratenon could not be completely sealed. Among 68 patients, 59 patients (86.8%) had a completely sealed paratenon—37 in group A and 22 in group B (*P* = 0.681). As mentioned, some patients underwent short leg cast immobilization. Specifically, 19 patients in group A and 6 patients in group B were treated with short leg casts (*P* = 0.068).

The correlations of patient characteristics and treatment details with clinical outcomes are shown in Table 3. A significant correlation was found between sex and VISA-A scores (*P* = 0.009), complete seal of paratenon and AOFAS scores (*P* = 0.031), length of leg cast and ATRS (*P* = 0.006). That is, male sex, complete seal of paratenon and short leg cast were related to better clinical outcomes.

**Table 3:** Correlation Between Patient Characteristics and Treatment Details with the Clinical Outcomes^a^

	VAS		AOFAS		VISA-A		Tegner		ATRS	
	Rank Correlation	*P* Value	Rank Correlation	*P* Value	Rank Correlation	*P* Value	Rank Correlation	*P* Value	Rank Correlation	*P* Value
Age	0.03	0.781	−0.01	0.968	−0.08	0.542	−0.06	0.629	−0.05	0.712
Sex	0.20	0.111	−0.20	0.108	**−0.31**	**0.009**	−0.10	0.441	−0.13	0.28
BMI	−0.10	0.453	−0.11	0.381	−0.13	0.292	0.10	0.440	0.02	0.898
Time to presentation (day)	−0.13	0.30	−0.12	0.333	−0.02	0.875	0.01	0.918	−0.04	0.726
Complete seal of paratenon	−0.10	0.428	**−0.26**	**0.031**	−0.04	0.739	0.02	0.845	−0.14	0.246
Length of leg cast	0.07	0.600	−0.22	0.078	−0.22	0.076	−0.07	0.588	**−0.33**	**0.006**

*VAS* visual analog scale; *AOFAS* American Orthopedic Foot and Ankle⁃Hindfoot Score; *VISA-A* the Victorian Institute of Sport Assessment⁃Achilles; *Tegner* the Tegner Activity Scale; *ATRS* the Achilles tendon total rupture score

^a^Boldface indicates statistical significance (*P* < .05)

## Discussion

The most important finding of the present study was that primary repair and augmented repair with a gastrocnemius turn-down flap for acute Achilles tendon rupture had comparable clinical outcomes. No significant between-group differences in pain level, tendon function and activity, isokinetic muscle strength, calf circumference, ability to perform everyday tasks, and time from surgery to rehabilitation were observed, in line with results from previous similar studies [23]. Despite the lack of clinical functional scores, Nyyssonen et al. [24] reported no significant differences in re-rupture rate or various subjective outcomes in 39 and 59 patients undergoing end-to-end repair and augmented repair, respectively. In a randomized controlled trial with a follow-up duration of more than 13 years, Heikkinen et al. [22] noted no significant differences in the Leppilahti score, isokinetic calf muscle strength, or Achilles tendon elongation of the patients undergoing primary repair and augmented repair, suggesting that augmented repair did not result in better objective clinical outcomes than end-to-end primary repair. In the present study of 68 patients, subjective and objective outcomes were both assessed, indicating no advantage of augmented repair over primary repair.

As pointed out before, the optimal treatment for acute Achilles tendon rupture has yet to be determined. Several recent, high-quality randomized controlled trials have indicated that conservative therapy can achieve comparable outcomes to surgical therapy for acute Achilles tendon rupture [5, 14, 19, 25]. Surgery reduced the risk for rerupture but increased the risk for other procedure-related complications [26]. However, conservative therapy cannot fully restore the function of the Achilles tendon because it retracts after rupture, resulting in delayed return to sport, persistent sensation of insecurity, and tendon elongation [6, 27]. Thus, surgical therapy remains to be an important option for patients with high functional demand [28]. Regarding open surgery, primary end-to-end repair is a simple operation involving smaller incisions [29]. Augmented repair aims to increase the strength of the Achilles tendon, facilitate its healing, and reduce the risk of re-rupture [22]. However, longer incision frequently leads to problems with wound healing, which may in turn result in tendon deformation [24]. Since no statistical differences of clinical outcomes were noted in the present study, there is no need to pursue augmented repair at the expense of longer incision and more complex procedure.

The patient-related factors that influence treatment outcomes after acute Achilles tendon rupture have drawn increasing attention in recent years, for example, age, BMI and sex differences [30]. Nevertheless, the questions of whether they play a role have been much debated [30–33]. Cramer et al. found a better clinical outcome for patients over 65 years than those between 40 and 65 years [31], while in the study by Arverud et al., over the age of 40 years was established as an independent negative predictor of outcome in patients with acute Achilles tendon rupture [33]. In the present study, no correlation was found between age and any clinical functional scores. One possible reason is that the study only included the patients with an age between 18 and 50 years, attenuating the effect of age increase on clinical outcomes. In our study, sex difference was found significantly correlated with VISA-A scores. The results are consistent with previous studies about sex-specific effects on the outcomes after acute Achilles tendon rupture, and similarly limited by small female sample size due to the low female-to-male ratio of Achilles tendon rupture.

In a preclinical study by Müller et al. [34], a preserved paratenon was found to contribute to Achilles tendon healing in the rat model. In the present study, we similarly found a significant correlation of complete seal of paratenon with AOFAS score, which has important implication for clinical practice. During surgical repair of acute Achilles tendon rupture, the paratenon should be sealed as completely as possible to reduce tendon adhesion and promote functional recovery.

As for leg casts, immobilization with a short leg cast in accordance with the principle of early enhanced recovery and provides greater comfort with adequate support. Mortensen et al. [35] conducted a prospective study comparing rehabilitation outcomes in patients receiving conventional postoperative management with a cast and patients allowed early restricted ankle motion (by wearing a below-the-knee brace). Similar to our results, the patients managed with early motion recovered faster and did not develop any complications.

In the present study, no serious complications in the follow-up period were reported in groups A or B, similar to previous studies [20, 27]. As Garabit et al. [29] did, we used absorbable sutures and attempted to completely seal the paratenon to reduce the risk of complications. All the patients, regardless of whether they were treated with long or short leg casts, followed a relatively conservative rehabilitation plan. Moreover, we reminded the patients to avoid forceful contact of the involved foot with the ground and to take care not to fall before they regained their weight-bearing walking abilities, because these are main factors leading to re-rupture of the Achilles tendon.

The strengths of this study are the relatively large sample size, a long follow-up duration, and the use of multiple types of subjective assessments of pain and tendon function—specifically, with five evaluation scales. Moreover, objective indicators of surgery effectiveness were also evaluated by using isokinetic testing and the measurement of postoperative calf circumference.

However, our study is subject to some limitations. Firstly, as a retrospective study, the evidence was less robust than that in a prospective study. A prospective randomized controlled study in future would provide greater evidence in determining best practice. Moreover, due to the too long distance to transport to our hospital and the relative long follow-up time, rate of loss to follow-up was relatively high (27.4%), which may underestimate the complications and cause bias. However, when comparing baseline data of two groups, no relevant differences were found. Finally, there was within-group heterogeneity that may induce confounding bias. Although there were no significant differences between groups regarding paratenon seal and length of leg cast, a future prospective study with more rigorous design is needed to validate the findings.

## Conclusion

In conclusion, the present study showed that augmented repair with a gastrocnemius turn-down flap provided no advantage over primary repair for the treatment of acute Achilles tendon rupture. After surgical treatment, females tended to had poorer outcomes, while complete seal of paratenon and short leg cast contributed to better results.

## Data Availability

The datasets used and/or analysed during the current study are available from the corresponding author on reasonable request.

## Abbreviations

VAS: Visual analogue scale
AOFAS: American Orthopaedic Foot and Ankle Society Ankle⁃Hindfoot Score
VISA-A: The Victorian Institute of Sport Assessment⁃Achilles
ATRS: The Achilles tendon total rupture score
Tegner: The Tegner Activity Scale
